# The efficacy of first and second immunotherapy exposure in patients with recurrent or metastatic cervical cancer

**DOI:** 10.1002/cam4.70204

**Published:** 2024-10-09

**Authors:** Mingxiu Ju, Baoyue Pan, Yongwen Huang, Yun Zhou, Jieping Chen, Huiling Xiang, Shije Xu, Siyu Chen, Chunyan Lan, Jundong Li, Min Zheng

**Affiliations:** ^1^ Department of Gynecology, State Key Laboratory of Oncology in South China, Guangdong Provincial Clinical Research Center for Cancer Sun Yat‐sen University Cancer Center Guangzhou P.R. China

**Keywords:** antiangiogenic therapy, immunotherapy, recurrent or metastatic cervical cancer

## Abstract

**Objective:**

Immunotherapy has led to changes in cervical cancer guidelines. Therefore, additional biomarkers to identify the ideal patient who would experience the most benefit may be important.

**Methods:**

We retrospectively collected 208 patients with R/M CC and recorded clinicopathologic information, peripheral blood markers and treatments to analyze the prognostic factors of clinical outcomes. Response rate comparison, univariate, and multivariate analyses were performed to assess the efficacy of different factors.

**Results:**

A total of 43.27% patients achieved objective responses, including 18 with complete response and 72 with partial response. Patients receiving first‐line immunotherapy had much higher objective response rate (ORR) than the remaining patients (53.8% vs. 34.8%, *p* = 0.006). CRP >3 ECOG ≥1 and recurrence in 6 months predicted shorter progression free survival (PFS). CRP >3, GLU >6.1 independently predicted unfavorable overall survival (OS). Compared with no antiangiogenic therapy, previous antiangiogenic therapy reduced the median OS by nearly 14 months. Immunotherapy rechallenge was still effective after first immunotherapy failure, and combined with dual‐immunotherapy or bevacizumab combined with chemoradiotherapy resulted in a 60.00% or 62.50% ORR, respectively. Patients with squamous cell carcinoma, with stable disease or objective response in the first immunotherapy or without chemotherapy in second immunotherapy had favorable clinical outcome.

**Conclusion:**

The baseline CRP levels in serum was an independent factor for PFS and OS of R/M CC patients treated with immunotherapy, and previous antiangiogenic therapy was associated with poor OS. Patients still show response to immunotherapy rechallenge and combined treatment with bevacizumab or candonilimab showed higher response rate than anti‐PD‐1 after immunotherapy failure.

## 1 INTRODUCTION

Despite cervical cancer screening and human papillomavirus vaccine promotion, there are still millions of new diagnoses and deaths from cervical cancer, ranking as the fourth most prevalent and deadliest cancer in women globally.^1^

The 5‐year survival rate in cervical cancer patients with recurrence was no more than 50% in previous studies.^2^, ^3^, ^4^ Chemotherapy based on platinum was the standard treatment for recurrent or metastatic cervical cancer (R/M CC) in the past three decades.^5^ Bevacizumab was also recommended to be combined with platinum‐based chemotherapy due to the results of the GOG 204 trial.^6^ However, fewer than 50% of patients show a response to bevacizumab^6^ and the cost of bevacizumab is prohibitive in low‐ and middle‐income countries with most cervical cancer occurrences.^7^ It has been reported that chemotherapy plus pembrolizumab is cost‐effective relative to chemotherapy plus bevacizumab.^8^

Immunotherapy has changed the treatment guidelines for cervical cancer. Pembrolizumab monotherapy resulted in a 13.3% overall response rate (ORR).^9^ Treatment with tyrosine kinase inhibitors (TKIs) combined with anti‐PD‐1 yielded an approximately 55% ORR in advanced disease with at least one prior line of treatment failure.^10^, ^11^ The latest results of KEYNOTE‐826 showed that pembrolizumab combined with chemotherapy increased the median overall survival (OS) by approximately 10 months.^12^ To further select the potential patients experiencing the most benefit from immunotherapy, several studies suggested that the pretreatment neutrophil‐to‐lymphocyte ratio, lactate dehydrogenase,^13^ squamous cell carcinoma, and a time to recurrence >6 months,^14^ serum C‐reactive protein (CRP)^15^ were independent factors associated with progression‐free survival (PFS). In addition, genetic alterations in *PIK3CA*, the PI3K‐AKT signaling pathway, TMB, and positive expression of PD‐L1 are all related to better clinical response.^11^, ^16^, ^17^ Metabolic related biomarkers have also been widely discussed. Hyperglycemia‐mediated immune dysfunction^18^ and diabetic cancer patients experienced less benefit from immunotherapy.^19^ High cholesterol is related to improved survival in patients treated with immunotherapy in the previous studies.^20^, ^21^

To date, the relationship between previous antiangiogenic treatment or metabolic factors and the response to immunotherapy has rarely been discussed in R/M CC. In non‐small cell lung cancer, antiangiogenic agents before immunotherapy did not show survival benefits in a meta‐analysis.^22^ The treatment response rate after immunotherapy progression in cervical cancer is also limited. Only one paper recently showed that rechallenge immunotherapy had minimal activity in cervical cancer.^23^

Thus, we performed a retrospective study to assess immunotherapy efficacy and prognostic factors in patients with R/M CC. Furthermore, we herein discuss the treatment choices after immunotherapy for patients with R/M CC.

## 2 METHODS

### 2.1 Patient selection and characteristics

We initially enrolled 356 patients diagnosed with cervical cancer who underwent immunotherapy between August 1, 2018, and September 30, 2022, at our hospital. Thirteen patients experienced fewer than 2 cycles, and 122 patients lacked at least two imaging assessments. Thirteen patients underwent immunotherapy when initially diagnosed with CC. Thus, 208 patients with R/M CC who experienced progression after initial treatment were ultimately enrolled in this study. The primary endpoint was OS, and the secondary endpoints included ORR, DCR, PFS. PFS2 was defined as time from the date of the immune therapy failure to the follow‐up deadline or the date of death. The immunotherapy agents collected for OS and PFS analysis included pembrolizumab, nivolumab, tislelizumab, camrelizumab, and sintilimab. The latter three antibodies were progressed by Chinese companies targeting PD‐1. They have been promoted into late‐stage studies and regulatory review in China.^24^ As for PFS2, cadonilimab, a PD‐1/CTLA4 bi‐specific antibody approved in China in June 2022 for use in patients with R/M CC who have progressed on or after platinum‐based chemotherapy,^25^ was also collected.

Demographics, tumor stage and histology, treatment agents, pretreatment lab values, adverse events, and survival outcomes were obtained. The follow‐up of this study was as of June 30, 2023. This study was approved by the Institutional Review Board of Sun Yat‐sen University Cancer Center and obtained an informed consent exemption due to its non‐interventional retrospective study. (B2023‐465‐01).

### 2.2 Assessment of antitumor response and adverse events

The antitumor response was classified as complete response (CR), partial response (PR), stable disease (SD), or progressive disease (PD), which was calculated based on computed tomography (CT), magnetic resonance imaging, or positron emission tomography/CT, according to the Response Evaluation Criteria in Solid Tumors (RECIST) v1.1. The objective response rate (ORR) was calculated as the proportion of patients who responded to immunotherapy. The disease control rate (DCR) was calculated by the proportion of patients without disease progression. Adverse events (AEs) were recorded based on the National Cancer Institute Common Terminology Criteria for Adverse Events (CTCAE) v4.0.

### 2.3 Cutoff of pretreatment lab values

The cutoff for the neutrophil‐to‐lymphocyte ratio was 5, which was obtained from several studies.^26^, ^27^ Hypercholesterolemia and hypertriglyceridemia were defined based on cutoff values of 5.2 mmol/L and 1.7 mmol/L of cholesterol and triglycerides in serum, respectively, according to the American Heart Association Guidelines.^28^ Low high‐density lipoprotein cholesterol, low low‐density lipoprotein cholesterol, high C reaction protein, low albumin, high blood sugar, and high lactate dehydrogenase were defined with values of 1.29 mmol/L, 2.2 mmol/L, 3 mg/L, 40 g/L, and 250 U/L in serum (representing the reference ranges of the population), respectively. Overweight was defined as ≥24 kg/m^2^ based on the Chinese Preventive Medicine Association Behavioral Health Section.^29^

### 2.4 Statistical analysis

Pearson's chi‐square test and continuity correction were carried out to analyze the relationships between the factors and response rate. The Kaplan–Meier method was used to plot the survival curves and the log‐rank test was used to analyze survival rates. Cox proportional hazards regression analysis was used to assess independent factors related to survival. *p* < 0.05 was regarded as statistically significant. Statistical analyses were performed using IBM SPSS v25.0 (IBM, Armonk, NY, U.S.A.).

## 3 RESULTS

### 3.1 Baseline characteristics

The baseline characteristics of these 208 patients with R/M CC are listed in Table 1. The median age was 53 years. Nearly one‐fourth of patients were diagnosed initially at early FIGO stage (I‐II) and three‐fourths of patients were diagnosed with squamous cell carcinoma. More than half of the patients underwent radical surgery and less than one‐third of the patients underwent radical radiation. Before immunotherapy, 29.32% of all patients used antiangiogenic agents (represented by bevacizumab). Most patients had metastasis outside the pelvis and 27.88% of patients had confined pelvic recurrence. The proportions of patients with pelvic hollow organ involvement, lung metastasis, and liver recurrence were 12.5%, 39.4%, and 11.06%, respectively. After initial treatment, 44.71% of patients with R/M CC underwent immunotherapy as first‐line treatment. Only 10% of patients met more than two lines of therapy before immunotherapy. Other treatments including chemotherapy, radiation therapy, and antiangiogenic treatment, were commonly combined to enhance the efficacy of immunotherapy. A total of 42.79% of patients had immunotherapy‐related AEs. Nearly one‐third of patients received more than 10 cycles of immunotherapy.

**TABLE 1:** Baseline patient characteristics.

Characteristics	Patients (*n* = 208)
Median age (year)	53 (24–72)
ECOG PS	
0	123 (59.13%)
1	71 (34.13%)
2	14 (6.73%)
FIGO stage	
I	40 (19.23%)
II	34 (16.34%)
III	84 (40.38%)
IV	23 (11.05%)
Histology	
Squamous cell carcinoma	157 (75.48%)
Adenocarcinoma	32 (15.38%)
Adenosquamous carcinoma	3 (1.44%)
Neuroendocrine carcinoma	14 (6.73%)
Initial treatment	
Radical surgery	131 (62.98%)
Radical radiotherapy	66 (31.73%)
Time to recurrence	
≤6 months	58 (27.88%)
>6 months	150 (72.12%)
Previous anti‐angiogenic treatment	
Yes	61 (29.32%)
No	145 (69.71%)
Previous bevacizumab	42 (20.19%)
Recurrence outside the pelvis	
Yes	150 (72.12%)
No	58 (27.88%)
Recurrence in bowel and bladder	26 (12.50%)
Recurrence in lung	82 (39.4%)
Recurrence in liver	23 (11.06%)
Time of immunotherapy	
First‐line	93 (44.71%)
Second‐line	94 (45.19%)
Third‐line or more	21 (10.10%)
Combined with therapeutic regimens	
Chemotherapy	130 (62.50%)
Radiation	34 (16.35%)
Anti‐angiogenic treatment	117 (56.25%)
irAE	89 (42.79%)
≥10 Cycles of immunotherapy	64 (30.76%)

Abbreviation: irAE, immune related adverse event.

### 3.2 Factors associated with outcomes of immunotherapy

Among all 208 patients, 90 patients (43.27%) obtained objective responses, including 18 with CR and 72 with PR. SD and PD were observed in 81, and 37 patients, respectively. The median duration of response (DOR), progression free survival (PFS), and OS were 13.23, 9.8, and 16.32 months, respectively.

To further investigate factors related to the outcomes of immunotherapy, we compared the ORR and DCR based on different subgroups (Table S1). The results revealed that patients with higher ECOG scores had lower ORR (*p* = 0.027) and DCR (*p* = 0.004). The DCRs for patients with confined pelvic recurrence, lung recurrence, and liver recurrence were 92.9%, 75.6%, and 65.2%, respectively. The time to use immune checkpoint inhibitors obviously influenced the efficacy (*p* = 0.006). The ORRs for patients receiving 1‐line immunotherapy and ≥2‐line immunotherapy were 53.8% and 34.8%, respectively. The appearance of immunotherapy related AEs indicated a higher DCR (90.7% vs. 77.4%, *p* = 0.017). Interestingly, previous antiangiogenic treatments, especially bevacizumab, had a poor effect on the DCR (73.8% vs. 85.5%, 71.4% vs. 84.9%). This gap was much more obvious when comparing ORR between patients with or without previous apatinib (17.6% vs. 45.5%).

**FIGURE 1:**
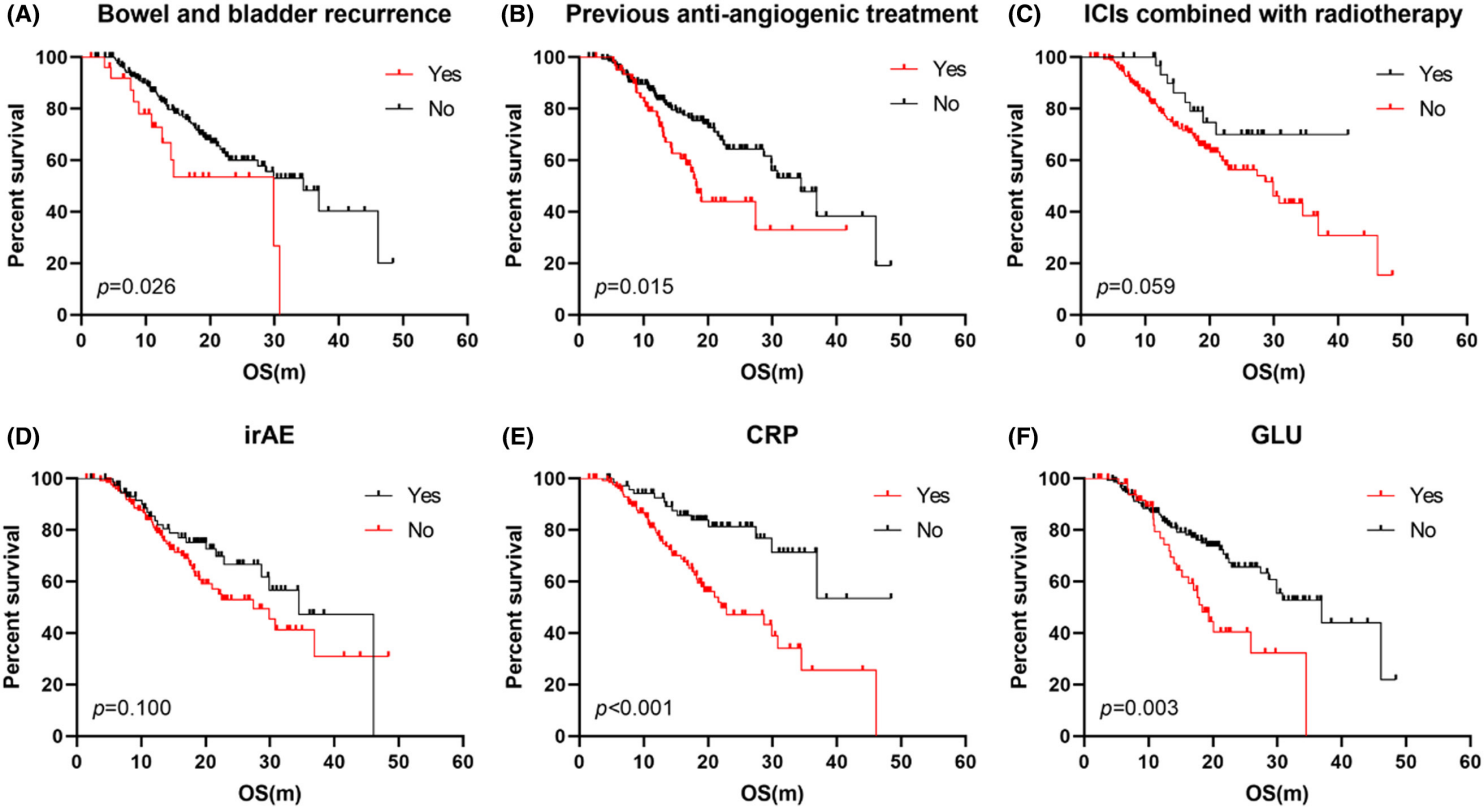
The Kaplan–Meier plots of univariate analysis of overall survival (A–F).

**FIGURE 2:**
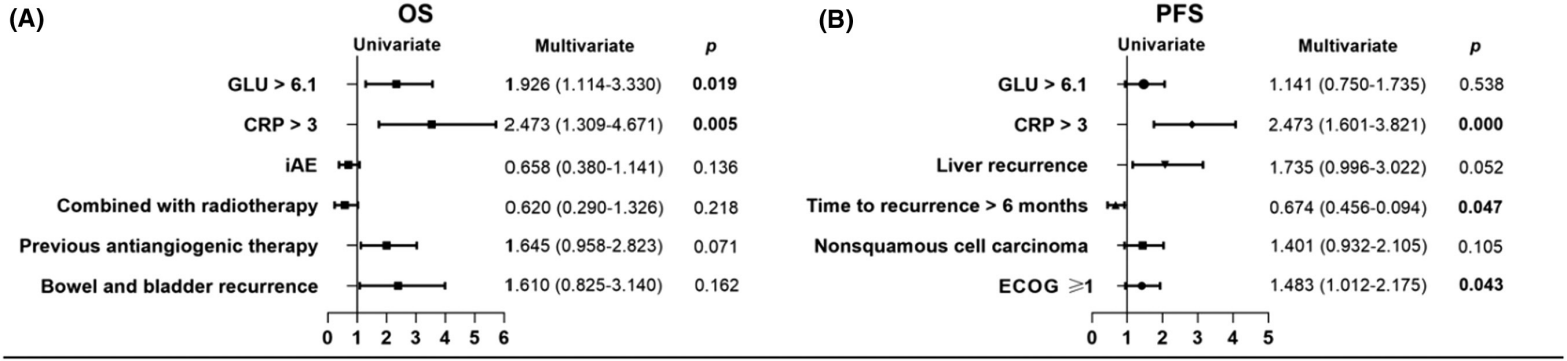
Forest plot for overall survival (A) and progression‐free survival (B) to immunotherapy according to factors selected by univariate analysis and their contributions in cox multivariate analysis.

Peripheral blood metabolic and inflammatory biomarkers, and clinical characteristics selected by the log rank test were further included to build the Cox proportional hazards regression to predict the OS and PFS in R/M CC treated with immuotherapy (Tables S2 and S3). In univariate analysis of OS, patients with bowel and bladder recurrence, without radiation therapy, without irAE, without previous antiangiogenic therapy, CRP >3 and GLU >6.1 were related to poor clinical outcomes (Figure 1). CRP >3 and GLU >6.1 were independent factors predicted an unfavorable OS. CRP >3, ECOG ≥1 and recurrence in 6 months predicted shorter PFS (Figure 2).

In addition, we found the median OS in the group without previous antiangiogenic therapy was 34.47 months compared to 20.57 months in the group with previous antiangiogenic therapy. Most of patients with previous antiangiogenic therapy underwent immunotherapy as second or more line treatment, whose resistance to antiangiogenic therapy likely reduce the immunotherapy efficacy.

### 3.3 AEs

All AEs were recorded in 168 patients (Table S4) and the most common AEs were: anemia (38.46%), hypothyroidism (18.75%) and ALT/AST elevation (11.06%). Immunotherapy‐related AEs leading to discontinuation were rare but a troublesome clinical problem with the need for comprehensive treatment. Insulin‐dependent diabetes and myositis hepatitis induced with checkpoint inhibitors were the main reasons for immunotherapy cessation.

### 3.4 Treatments following immunotherapy progression

Thirty seven patients with detailed treatment and imaging assessment after immunotherapy progression were collected to perform further therapy response comparisons. At the end of previous immunotherapy, only 4 patients achieved PR, 4 patients achieved SD, and 29 patients were assessed as having PD. Among them, 29 patients continued to use immunotherapy with changing combined agents (Table 2). In detail, 19 patients still treated with combined anti‐PD‐1 treatment and 10 patients underwent dual‐immunotherapy. The other 8 patients received chemoradiotherapy +antiangiogenic therapy (C + A). C + A (62.50%) revealed a much higher ORR advantage than immunotherapy rechallenge (41.28%). Notably, cadonilimab showed more obvious disease remission in patients with immunotherapy progression than anti‐PD‐1 (60.00% vs. 31.58%).

**TABLE 2:** Following treatments after immunotherapy progression.

	Immunotherapy rechallenge			Chemotherapy/Radiation combined with antiangiogenic therapy (*n* = 8)
	All (*n* = 29)	Anti‐PD1 (*n* = 19)	Anti‐PD1/CTLA4 (*n* = 10)	
CR	1	0	1	1
PR	11	6	5	4
SD	11	9	2	0
PD	6	4	2	3
ORR	41.38%	31.58%	60.00%	62.50%
DCR	79.31%	78.95%	80.00%	62.50%

To further investigate the efficacy of second immunotherapy, we summarized the baseline characteristics (Table 3) and analyzed the prognostic factors of these patients (Table 4). Nearly one third of patients were nonsquamous cell carcinoma and half of patients had lung recurrence. We found nonsquamous cell carcinoma and second immunotherapy combined with chemotherapy related to shorter PFS2. In addition, the response to previous immunotherapy directly affected the efficacy of immunotherapy rechallenge. Patients reached controlled disease status in first immunotherapy had longer survival in the following immunotherapy.

**TABLE 3:** Baseline characteristics of patients with immune rechallenge.

Characteristics	Patients (*n* = 29)
Median age (year)	53 (24–67)
Histology	
Squamous cell carcinoma	20 (68.97%)
Other	9 (31.04%)
Recurrence	
Outside the pelvis	20 (68.97%)
Lung	14 (48.28%)
Previous immunotherapy	
Combined with chemotherapy	17 (58.62%)
Combined with antiangiogenic treatment	13 (44.83%)
Response of previous immunotherapy	
Objective response	10 (34.48%)
Controlled disease	9 (31.04%)
Progressive disease	10 (34.48%)
Immune rechallenge	
Combined with chemotherapy	13 (44.83%)
Combined with antiangiogenic treatment	17 (58.62%)
Anti‐PD‐1	19 (65.52%)
Anti‐PD‐1/CTLA‐4	10 (34.48%)

**TABLE 4:** Univariate and multivariate analysis of PFS2.

	Univariate			Multivariate		
	*p*	HR	95% CI	*p*	HR	95% CI
Histology						
Others versus squamous cell carcinoma	**0.026**	3.986	1.193–13.320	**0.041**	3.658	1.054–12.701
Recurrence outside the pelvis	0.734	1.311	0.274–6.264			
Lung recurrence	0.975	0.982	0.327–2.950			
Previous immunotherapy						
Combined with chemotherapy	0.087	3.062	0.851–11.021			
Combined with antiangiogenic therapy	0.433	0.625	0.193–2.023			
Response of previous immunotherapy						
Without progressive disease	**0.058**	0.356	0.122–1.036	0.329	0.560	0.175–1.792
Immune rechallenge						
Combined with chemotherapy	**0.057**	3.217	0.968–10.691	0.224	2.364	0.590–9.474
Combined with antiangiogenic treatment	0.894	0.928	0.307–2.807			
Anti‐PD‐1 versus Anti‐PD‐1/CTLA‐4	0.206	2.643	0.587–11.900			

*p* = 0.026, 0.041, *p* < 0.05 means there is a significant difference. *p* = 0.057 and 0.058 are close to 0.05, which means they have a high correlation.

## 4 DISCUSSION

Our study confirmed that immunotherapy led to a high response rate (43.27%) for patients with R/M CC. ECOG score, previous apatinib use, first‐line immunotherapy and dexamethasone use were significantly related to ORR. CRP >3 and GLU >6.1 predicted unfavorable OS. Further analysis suggested that TKI after previous bevacizumab reversed the negative effect of previous antiangiogenic therapy on immunotherapy. Finally, immunotherapy rechallenge was efficacy in R/M CC patients. C + A or dual‐immunotherapy was found to be a more efficacy treatment for immunotherapy progression.

Numerous mono‐immunotherapies or immunotherapy combinations are under evaluation for R/M CC. The status of immunotherapy was recommended from mono‐therapy for second‐line therapy, combined with apatinib/anlotinib for second‐line therapy, to combined with chemotherapy for first‐line therapy.^9^, ^10^, ^11^, ^12^, ^30^ Immunotherapy for first‐line therapy in our results yielded an ORR of 53.76% with a DCR of 86.02% and a median PFS of 11.2 months, which were better than those of second‐line therapy. A high ECOG score was associated with a poor clinical outcome and was commonly reported in other tumors.^31^, ^32^ Nonsquamous tumors were an unfavorable factor for PFS in our study but had no relationship with OS. The effect of histology on the efficacy of immunotherapy was variable and inconclusive in different studies.^11^, ^33^, ^34^, ^35^ Peripheral blood metabolic and inflammatory biomarkers have been widely discussed as presenting an easy method to predict survival. CRP is commonly utilized as an inflammatory marker and has been extensively investigated as a prognostic and predictive biomarker for various malignancies. CRP has been employed to evaluate the prognosis of patients undergoing such treatments. In numerous cancer types, CRP levels serve as a robust indicator for predicting the outcomes of immunotherapy. Elevated CRP levels are positively correlated with poorer OS and PFS.^36^, ^37^, ^38^ The cutoff of CRP in this study was based on the reference value instead of the median value^39^ because median value was instable and variable in different populations. CRP >3 was the only factor strongly associated with PFS and OS. In addition, high blood sugar is believed to be linked to immune abnormalities, as glucose metabolism plays a crucial role in the proliferation, differentiation, and function of immune cells, as well as in shaping immune responses.^40^, ^41^ we found that high blood sugar predicted poor OS. Additionally, the influence of hyperglycemia on the effectiveness of immunotherapy has been demonstrated in other studies.^19^, ^42^ However, markers related to blood lipids show no effect on PFS or OS in our results.

Apatinib and anlotinib, TKIs of VEGFR2 and VEGFRs, retrospectively or prospectively showed approximately 15% and 25% ORR in R/M CC with chemotherapy failure.^43^, ^44^, ^45^ After prior antiangiogenic therapy for R/M CC was associated with a lower response rate, we could not exclude the possibility that progression after prior antiangiogenic therapy was just a marker for treatment‐resistant cancer. In our study, we found that the median OS of the group that had not previously received anti‐angiogenic therapy was 34.47 months, while the median OS of the group that had previously received anti‐angiogenic therapy was 20.57 months. This difference may be attributed to the development of resistance to anti‐angiogenic therapy during initial treatment, which results in changes in the local microenvironment. The research demonstrated that the constructed mouse model exhibited adaptive resistance to VEGFR, and sequencing results indicated a significant up‐regulation of CD5L.^46^ The key role of CD5L in the immune microenvironment has been investigated in multiple studies.^47^, ^48^, ^49^ Additionally, intratumoral hypoxia and stable hypoxia‐inducible factor 1a (HIF‐1a) are characteristics of the microenvironment that emerge following resistance to anti‐angiogenic drugs,^50^ which in turn affects the efficacy of immunotherapy.^51^, ^52^, ^53^ The mechanisms underlying this aspect warrant further investigation in future studies. But we found that TKI combined immunotherapy after previous bevacizumab failure demonstrated a similar median OS when compared to patients without previous antiangiogenic treatment (17.61 vs. 18.4 months). This revealed that changing VEGFR inhibitors from VEGF inhibitors overcame the failure of bevacizumab treatment, which had been reported in renal cell carcinoma.^54^

Anemia and hypothyroidism were the most common AEs and irAEs in our study and previous studies,^55^, ^56^ respectively. Discontinuation caused by grade 3–4 AEs was rare, and immunotherapy was safe for R/M CC.

We also discussed the efficacy of immunotherapy rechallenge. About 10%–30% patients displayed a reduction and 70% patients showed SD in the post‐progression period with pembrolizumab in the latest study.^57^ Second immunotherapy exposure had limited studies in cervical cancer and the latest paper included four patients who underwent anti‐PD‐1 after anti‐PD‐1: 3 patients with PD and one patient with SD.^23^ In our results, for patients with PD during treatment with a PD‐1 inhibitor, although the number of cases was limited, an objective response from anti‐PD‐1 rechallenge was still achieved in about 30% patients and nearly 80% patients had controlled disease in the post‐progression period. It indicated that subsequent lines of immunotherapy has certain effectiveness in R/M CC patients. In addition, as reported in melanoma,^58^ escalation to a treatment combining a PD‐1 inhibitor with inhibitors of cytotoxic T‐lymphocyte‐associated protein 4 (CTLA‐4) obtained the objective response again. Our results also showed that anti‐PD‐1/CTLA4 had higher ORR than anti‐PD‐1. Further analysis showed that the response to the first immunotherapy exposure influenced the PFS of patients treated with second immunotherapy, and nonsquamous tumors were also related with poor clinical outcomes in second immunotherapy.

In contrast to second immunotherapy exposure, chemotherapy combined with bevacizumab resulted in a 62.5% ORR, suggesting the potential of retreatment with chemotherapy and bevacizumab, which was reported in advanced lung cancer.^59^ Fundamental research has also reported that anti‐PD‐1 therapy reshapes the blood vessels in tumors and enhances the subsequent efficacy of antiangiogenic therapy.^60^ Further survival analysis showed that anti‐PD‐1 related to shorter remission comparted to anti‐PD‐1/CTLA4 or bevacizumab combined with chemoradiotherapy, although without statistically significance (Figure S1, *p* = 0.093).

There were several objective limitations of this study. First, this was a mono‐center retrospective study with no control group for comparison; thus, selection bias was inevitable. A significant number of our patients were transferred from lower‐level hospitals due to their complex conditions. Additionally, the retrospective analysis is confined to a single center. While this study demonstrates the effectiveness of the treatment, it may also lead to a reduced ORR. Second, the expression of PD‐L1 and genetic testing results were not completely collected due to the expenses. The efficacy of PD‐L1 expression and PIK3CA mutation remained unclear in this large population. Finally, the small sample size in the subgroup analysis for immunotherapy rechallenge led to findings that need more investigations and prospective clinical trials for confirmation.

Our data showed that immunotherapy was a rational treatment for R/M CC. Although the time to use immunotherapy did not affect OS, first‐line immunotherapy showed a higher ORR. CRP >3, GLU >6.1 were independently related to poor OS. Immunotherapy rechallenge might be a potential treatment after immunotherapy failure, but dualimmunotherapy or antiangiogenic therapy combined with chemotherapy showed higher response rate.

## AUTHOR CONTRIBUTIONS

**Mingxiu Ju:** Formal analysis (lead); investigation (lead); writing – original draft (lead). **Baoyue Pan:** Formal analysis (equal); investigation (equal); writing – original draft (equal). **Yongwen Huang:** Formal analysis (equal); investigation (equal); writing – original draft (equal). **Yun Zhou:** Methodology (equal). **Jieping Chen:** Funding acquisition (equal). **Huiling Xiang:** Data curation (lead). **Shije Xu:** Visualization (equal). **Siyu Chen:** Visualization (equal). **Chunyan Lan:** Conceptualization (equal); writing – review and editing (equal). **Jundong Li:** Conceptualization (equal); visualization (equal). **Min Zheng:** Conceptualization (lead); funding acquisition (lead); visualization (lead).

## CONFLICT OF INTEREST STATEMENT

The authors report no conflict of interest.

## Supporting information


DATA S1:

## Data Availability

The datasets generated and analyzed during the current study have not been made public in order to protect the clinical information of the patients. However, it is available from the corresponding author upon reasonable request.
