# Supplementary material for: The efficacy of first and second immunotherapy exposure in patients with recurrent or metastatic cervical cancer
Source: Cancer Med. 2024 Oct 9;13(19):e70204. doi: 10.1002/cam4.70204 (PMC11462590; doi:10.1002/cam4.70204)
Supplement: Supplementary file 1 — DATA S1: [file CAM4-13-e70204-s001.docx]

**Table S1. Demographic and tumor characteristics based on response**

| **Characteristics** | **ORR** | | | **DCR** | | |
| --- | --- | --- | --- | --- | --- | --- |
|  | **%** | | ***p*** | **%** | | ***p*** |
| **Age (year)** |  | 0.690 | |  | 0.646 | |
| > 50 | 42.2% |  | | 81.3% |  | |
| ≤ 50 | 45.0% |  | | 83.8% |  | |
| **ECOG PS** |  | **0.027** | |  | **0.004** | |
| 0 | 49.6% |  | | 88.6% |  | |
| ≥ 1 | 34.1% |  | | 72.9% |  | |
| **FIGO stage** |  | 0.570 | |  | 0.598 | |
| I-II | 44.6% |  | | 83.8% |  | |
| III-IV | 40.4% |  | | 80.7% |  | |
| **Histology** |  | 0.069 | |  | 0.252 | |
| Squamous cell carcinoma | 47.4% |  | | 84.6% |  | |
| Others | 32.7% |  | | 77.6% |  | |
| **Time to recurrence** |  | 0.513 | |  | 0.278 | |
| ≤ 6 months | 39.7% |  | | 84.0% |  | |
| > 6 months | 44.7% |  | | 77.6% |  | |
| **Previous** **anti-angiogenic treatment** |  | 0.152 | |  | **0.045** | |
| Yes | 36.1% |  | | 73.8% |  | |
| No | 46.9% |  | | 85.5% |  | |
| **Previous bevacizumab** |  | 0.683 | |  | **0.041** | |
| Yes | 40.5% |  | | 71.4% |  | |
| No | 44.0% |  | | 84.9% |  | |
| **Previous apatinib** |  | **0.026** | |  | 0.518 | |
| Yes | 17.6% |  | | 76.5% |  | |
| No | 45.5% |  | | 82.7% |  | |
| **Recurrence outside the pelvis** |  | 0.229 | |  | **0.013** | |
| Yes | 40.7% |  | | 78.0% |  | |
| No | 50.0% |  | | 92.9% |  | |
| **Recurrence in bowel and bladder** | 50.0% | 0.459 | | 96.2% | 0.087 | |
| **Recurrence in lung** | 41.5% | 0.672 | | 75.6% | **0.045** | |
| **Recurrence in liver** | 30.4% | 0.188 | | 65.2% | **0.049** | |
| **Time of immunotherapy** |  | **0.006** | |  | 0.196 | |
| First-line | 53.8% |  | | 86.0% |  | |
| ≥ Second-line | 34.8% |  | | 79.1% |  | |
| **Dexamethasone use** |  | **0.036** | |  | 0.722 | |
| Yes | 53.7% |  | | 81.6% |  | |
| No | 38.3% |  | | 83.6% |  | |
| **Combined with therapeutic regimens** |  |  | |  |  | |
| Chemotherapy | 44.6% | 0.613 | | 83.1% | 0.674 | |
| Radiation | 52.9% | 0.213 | | 85.3% | 0.607 | |
| Anti-angiogenic treatment | 38.5% | 0.113 | | 81.2% | 0.664 | |
| **irAE** |  | 0.185 | |  | **0.017** | |
| Yes | 49.3% |  | | 90.7% |  | |
| No | 39.8% |  | | 77.4% |  | |

**Table S2. Univariate and multivariate analysis of OS**

|  | Univariate | | | Multivariate | | |
| --- | --- | --- | --- | --- | --- | --- |
|  | *p* | HR | 95% CI | *p* | HR | 95% CI |
| Age (> 50 *vs*. ≤ 50) | 0.882 | 0.964 | 0.590–1.575 |  |  |  |
| ECOG (≥1 *vs*. 0) | 0.101 | 1.505 | 0.923-2.452 |  |  |  |
| FIGO stage |  |  |  |  |  |  |
| I+II *vs*. III+IV | 0.976 | 0.992 | 0.601-1.637 |  |  |  |
| Histology |  |  |  |  |  |  |
| Others *vs.* squamous cell carcinoma | 0.146 | 1.469 | 0.874-2.471 |  |  |  |
| Time to recurrence (> 6 vs. ≤ 6 months) | 0.658 | 0.888 | 0.526-1.500 |  |  |  |
| Bowel and bladder recurrence | **0.026** | 2.088 | 1.090-3.997 | 0.162 | 1.610 | 0.825-3.140 |
| Recurrence outside the pelvis | 0.996 | 1.001 | 0.558–1.706 |  |  |  |
| Lung recurrence | 0.844 | 0.953 | 0.587-1.546 |  |  |  |
| Liver recurrence | 0.227 | 1.544 | 0.763-3.127 |  |  |  |
| Previous anti-angiogenic therapy | **0.015** | 1.848 | 1.125-3.035 | 0.071 | 1.645 | 0.958-2.823 |
| First-line therapy with ICIs | 0.691 | 1.104 | 0.679-1.794 |  |  |  |
| Dexamethasone use | 0.616 | 0.875 | 0.519-1.474 |  |  |  |
| ICIs combined with chemotherapy | 0.145 | 0.700 | 0.433-1.132 |  |  |  |
| ICIs combined with radiotherapy | **0.059** | 0.491 | 0.234-1.027 | 0.218 | 0.620 | 0.290-1.326 |
| ICIs combined with anti-angiogenic therapy | 0.326 | 1.275 | 0.785-2.071 |  |  |  |
| iAE (yes *vs*. no) | **0.100** | 0.649 | 0.388-1.086 | 0.136 | 0.658 | 0.380-1.141 |
| NLR (≥5 *vs*. <5) | 0.171 | 1.421 | 0.859-2.352 |  |  |  |
| CRP (> 3 *vs*. ≤ 3) | **0.000** | 3.158 | 1.741-5.730 | 0.005 | 2.473 | 1.309-4.671 |
| ALB (> 40 *vs*. ≤ 40) | 0.357 | 0.780 | 0.460-1.323 |  |  |  |
| BMI (≥ 24 *vs*. < 24) | 0.659 | 1.121 | 0.675–1.863 |  |  |  |
| CHO (> 5.2 *vs*. ≤ 5.2) | 0.700 | 0.896 | 0.514-1.563 |  |  |  |
| TG (> 1.7 *vs*. ≤ 1.7) | 0.865 | 0.953 | 0.545-1.664 |  |  |  |
| HDL-C (> 1.7 *vs*. ≤ 1.7) | 0.243 | 1.341 | 0.819-2.193 |  |  |  |
| LDL-C (> 1.7 *vs*. ≤ 1.7) | 0.193 | 0.720 | 0.439-1.181 |  |  |  |
| GLU (> 6.1 *vs*. ≤ 6.1) | **0.003** | 2.151 | 1.296-3.570 | 0.019 | 1.926 | 1.114-3.330 |
| LDH (> 250 *vs*. ≤ 250) | 0.170 | 1.665 | 0.803–3.452 |  |  |  |

ICIs: immune checkpoint inhibitors.

**Table S3.** **Univariate and** **multivariate analysis of PFS**

|  | Univariate | | | Multivariate | | |
| --- | --- | --- | --- | --- | --- | --- |
|  | *p* | HR | 95% CI | *p* | HR | 95% CI |
| Age (> 50 *vs*. ≤ 50) | 0.449 | 0.871 | 0.608–1.247 |  |  |  |
| ECOG (≥1 *vs*. 0) | **0.098** | 1.352 | 0.946-1.932 | 0.043 | 1.483 | 1.012-2.175 |
| FIGO stage |  |  |  |  |  |  |
| I+II *vs*. III+IV | 0.152 | 1.333 | 0.900-1.976 |  |  |  |
| Histology |  |  |  |  |  |  |
| Others *vs.* squamous cell carcinoma | **0.122** | 1.365 | 0.920-2.023 | 0.105 | 1.401 | 0.932-2.105 |
| Time to recurrence (> 6 vs. ≤ 6 months) | **0.017** | 0.635 | 0.437-0.923 | 0.047 | 0.674 | 0.456-0.994 |
| Bowel and bladder recurrence | 0.460 | 1.227 | 0.713-2.113 |  |  |  |
| Recurrence outside the pelvis | 0.545 | 1.134 | 0.755–1.703 |  |  |  |
| Lung recurrence | 0.815 | 1.044 | 0.730-1.492 |  |  |  |
| Liver recurrence | **0.012** | 1.906 | 1.153-3.152 | 0.052 | 1.735 | 0.996-3.022 |
| Previous anti-angiogenic therapy | 0.142 | 1.327 | 0.909-1.937 |  |  |  |
| First-line therapy with ICIs | 0.260 | 1.228 | 0.859-1.754 |  |  |  |
| Dexamethasone use | 0.284 | 0.809 | 0.549-1.192 |  |  |  |
| ICIs combined with chemotherapy | 0.733 | 0.939 | 0.652-1.351 |  |  |  |
| ICIs combined with radiotherapy | 0.183 | 0.716 | 0.438-1.171 |  |  |  |
| ICIs combined with anti-angiogenic therapy | 0.880 | 0.973 | 0.682-1.389 |  |  |  |
| iAE (yes *vs*. no) | 0.156 | 0.764 | 0.527-1.108 |  |  |  |
| NLR (≥ 5 *vs*. < 5) | 0.191 | 1.285 | 0.882-1.872 |  |  |  |
| CRP (> 3 *vs*. ≤ 3) | **0.000** | 2.675 | 1.757-4.074 | 0.000 | 2.473 | 1.601-3.821 |
| ALB (> 40 *vs*. ≤ 40) | 0.602 | 0.896 | 0.593-1.355 |  |  |  |
| BMI (≥ 24 *vs*. < 24) | 0.269 | 1.241 | 0.847–1.818 |  |  |  |
| CHO (> 5.2 *vs*. ≤ 5.2) | 0.473 | 0.859 | 0.568-1.300 |  |  |  |
| TG (> 1.7 *vs*. ≤ 1.7) | 0.337 | 0.806 | 0.519-1.252 |  |  |  |
| HDL-C (> 1.7 *vs*. ≤ 1.7) | 0.893 | 1.026 | 0.704-1.497 |  |  |  |
| LDL-C (> 1.7 *vs*. ≤ 1.7) | 0.240 | 0.802 | 0.554-1.159 |  |  |  |
| GLU (> 6.1 *vs*. ≤ 6.1) | **0.097** | 1.392 | 0.942-2.058 | 0.538 | 1.141 | 0.750-1.735 |
| LDH (> 250 *vs*. ≤ 250) | 0.448 | 1.276 | 0.679–2.398 |  |  |  |

**Table S4. Treatment-related adverse events**

|  | Any Grade | Grade3-4 | Lead to treatment discountinuation |
| --- | --- | --- | --- |
|  | n | n | n |
| Any | 168 | 16 |  |
| Anemia | 80 | 1 |  |
| Hypothyroidism | 39 | 0 |  |
| ALT/AST elevation | **23** | 3 | 3 |
| Thrombocytopenia | 20 | 5 |  |
| Hypertension | **20** | 2 |  |
| Hand-foot syndrome | 19 | 0 |  |
| Neutropenia | 18 | 2 |  |
| Rash | 14 | 0 |  |
| High creatinine | 11 | 3 | 3 |
| Pain | 11 | 3 | 3 |
| Fatigue | 10 | 0 |  |
| Anorexia | 8 | 0 |  |
| Diarrhea | 7 | 0 |  |
| Nausea or vomiting | 7 | 0 |  |
| Hyperthyroidism | 7 | 0 |  |
| Headache/dizziness | 6 | 0 |  |
| Hyperglycemia | 5 | 1 | 1 |
| Hoarseness | 4 | 0 |  |
| Dental ulcer | 4 | 0 |  |
| Hypoalbuminemia | 4 | 0 |  |
| Constipation | 3 | 0 |  |
| Pneumonitis | 2 | 0 |  |
| Myocarditis | 2 | 0 |  |
| Creatine phosphokinase elevation | 2 | 1 | 1 |
| Fistula | 2 | 1 | 1 |
| Fever | 2 | 0 |  |


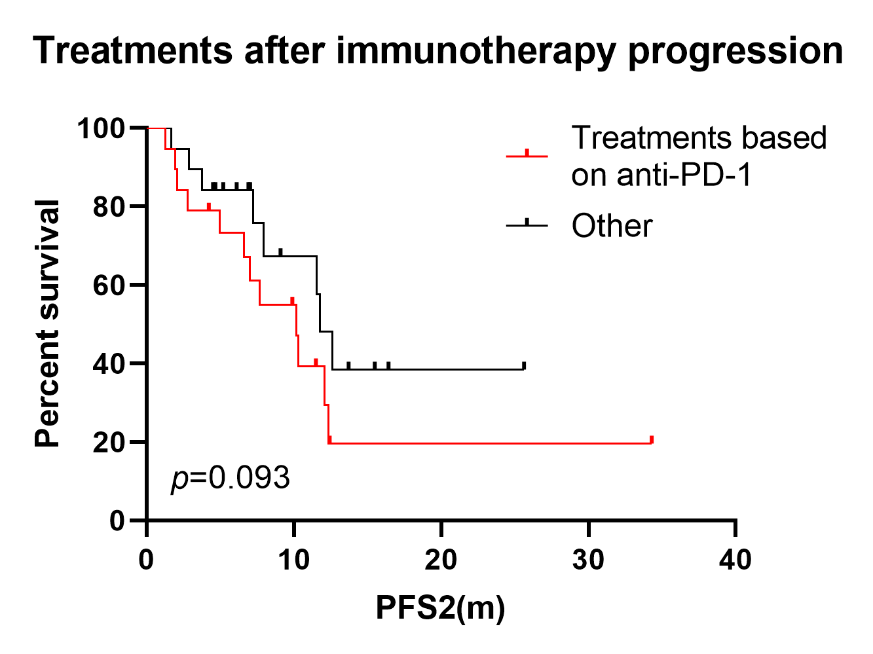


Supplemental figure 1: Kaplan–Meier curves of progression free survival for the patients with immunotherapy progression according to different treatment.
